# Flavonoids on the Frontline against Cancer Metastasis

**DOI:** 10.3390/cancers15164139

**Published:** 2023-08-17

**Authors:** Sarah Eltahir, Aamir Ahmad

**Affiliations:** 1 Translational Research Institute, Academic Health System, Hamad Medical Corporation, Doha 3050, Qatar; seltahir00@gmail.com; 2 Dermatology Institute, Academic Health System, Hamad Medical Corporation, Doha 3050, Qatar; 3 Department of Dermatology and Venereology, Rumailah Hospital, Hamad Medical Corporation, Doha 3050, Qatar; 4 Department of Anesthesiology and Perioperative Medicine, University of Alabama, 901 19th St. South, Birmingham, AL 35294, USA

Metastasis is the leading cause of death in cancer patients [1]. The act of invading and forming new microenvironments in distant tissues is the main feature of metastasis and indicates advanced disease. Flavonoids are natural substances found in fruits and vegetables and are known to possess anti-inflammatory, anticancer, antioxidant, and antimutagenic effects, securing health benefits [2]. Flavonoids act by regulating multiple signaling pathways during cancer initiation and promotion, cancer invasion, and metastasis formation. In combination with anticancer agents, flavonoids increase their efficacy and effectively reduce mortality among cancer patients [3,4].

Metastasis starts with the migration of cancer cells from the primary tumor to secondary lesions. The invasion–metastasis cascade starts with local invasion from the primary tumor leading to cancer cells to intravasate and adapt to survive in the blood circulation. Once obtaining this property, the cells then move to distant areas in the body and extravasate, causing micrometastasis and eventually metastasis colonization. Several factors determine metastasis, a few to mention are cancer and non-cancer cells interactions, biochemistry and biomechanics of the extracellular microenvironment, epigenetics, and intertumoral microbiome [5]. Chronic inflammation has been proven to affect tumors by increasing their development, progression, and increase therapy resistance. Tumor cells can produce cytokines which inhibit an antitumor immune response [6]. Epithelial–mesenchymal transition (EMT) is a process whereby cells become more motile and invasive, and this process plays an important role in cancer metastasis [7,8,9].

One of the goals of anticancer therapeutics is the prevention of metastasis in patients; however, metastatic cancer cells are resistant to therapies, owing to which, most patients undergoing chemotherapy often become resistant to treatment, leading to the reoccurrence of disease and metastasis. Liskova and co-authors discuss several flavonoids for their anticancer effects and the ability to block cancer metastasis [10]. They specifically mention six subtypes in their review article. The first subtype is Flavones, and the discussion starts with Apigenin, which has been found to be effective in prostate cancer cells, colon cancer cells, breast cancer cells, and melanoma cell lines. In all these cancer models, apigenin inhibited metastasis, cell migration and invasion, and EMT, specifically in both prostate cancer cells and colon cancer cells, and specifically decreased stem cell properties in breast cancer cells [10]. Another compound, Luteolin, when tested on squamous carcinoma cells, colorectal cancer cells, and melanoma cells, effectively decreased metastasis and cell invasion. Wogonin, acting on breast cancer cells and hepatocarcinoma cells, was seen to reduce migration and invasion, and when acting on human osteosarcoma cells, it reduced the stem-cell-like traits of the cancer cells. Wogonosides was found to be effective in breast cancer cells, reducing cancer metastasis and cell invasion. Hispudulin reduced the rate of metastasis in colorectal cancer cells and reduced migration, invasion, and metastasis in hepatocellular carcinoma cell lines. Pectolinarigenin decreased migration and invasion in colorectal cancer cells and decreased migration, invasion, and metastasis in breast cancer cells. Cirsiliol and Vicenin II reduced EMT in metastatic melanoma cells and lung adenocarcinoma cells, respectively. Oroxylin A effectively reduced tumor metastasis in oral squamous cell carcinoma cells. Scutellarin, acting on bladder cancer cells, was found to decrease migration, invasion, and EMT. Thus, the individual flavones [11,12,13] seem to exert anticancer effects via many mechanisms in different cancers, as reviewed by Liskova et al. [10].

The second flavonoids subtype mentioned are flavonols made up of Quercetin, Myricetin, Kaempferol, Fisetin, and Morin hydrate [14,15,16,17,18]. Quercetin was found to be effective in multiple cancers such as gastric cancer, non-small cell lung cancer, colorectal adenocarcinoma cells, melanoma, and prostate cancer. Quercetin reduced migration and invasion in gastric cancer and reduced migration, invasion, and cell metastatic abilities in non-small cell lung cancer. In colorectal adenocarcinoma, there was a reduced EMT, and in melanoma cells, quercetin reduced metastasis, invasion, and migration. Myricetin reduced migration, metastasis, and invasion in breast cancer cells and prostate cancer cells, while also reducing metastasis in cholangiocarcinoma cells. Kaempferol decreased migration and invasiveness in renal cancer cells. In breast cancer cells, the flavonol decreased tumor growth and metastasis. Fisetin affected non-small cell lung carcinoma cells by decreasing the migration, invasion of cells, and reducing the stem-cell-like properties of the cells. Fisetin also reduced cell mobility, migration, and invasion in human osteosarcoma cells. Morin hydrate reduced EMT in prostate cancer cells, reduced invasion and increased MET in melanoma cells, and reduced metastasis in breast cancer cells.

Flavanones [19,20,21] are another subtype of flavonoids. 2′-Hydroxyflavanone is the first mentioned flavanone in the article by Liskova et al. [10] and has proven to be effective against prostate cancer cells, reducing migration, invasion, and EMT. Liquiritigenin reduced invasion and EMT in colorectal cancer cells. Eriodictyol decreased growth and metastasis in glioma cells. Naringenin reduced migration, invasion, and EMT in both glioblastoma and prostate cancer cells. Taxifolin, acting on breast cancer cells, reduced migration, invasion, and metastasis while simultaneously increasing MET. Flavanol EGCG is effective against multiple cancers, as has been exhaustively reviewed [22,23,24]. Isoflavonoids include the well-studied genistein [25,26] which has been shown to decrease metastatic potential in prostate cancer cells and colorectal cancer cells, while also reducing migration, invasion, and EMT in ovarian cancer cells. Genistein, luteolin, and quercetin all worked together to reduce prostate stem cell antigen (PSCA), which is initially overexpressed in prostate cancer. Delphinidin [27,28] is the only flavonoid representing the anthrocynadins subtype. In colorectal cancer, delphinidin decreased migration, invasion, metastasis, and EMT, and in both osteosarcoma cells and hepatocellular carcinoma cells, delphinidin reduced EMT. Chalcones are the last-mentioned subtype consisting of isoliquiritigenin and phloretin. Isoliquiritigenin affected ovarian cancer cells by reducing EMT and gastric cancer cells by decreasing metastasis. Phloretin acted on cervical cancer cells and effectively reduced migration, invasion, and EMT.

Flavonoids undergo metabolism in the small and large intestine where they are catabolized into smaller molecules. Phase I metabolism is then initiated in the epithelium, and the metabolites travel to the liver where phase I and II metabolism occurs. Most flavonoids, after metabolism, have reduced bioavailability; however, a small percentage of flavonoids have a higher bioavailability and bioactivity [29,30]. It has been proven that multiple factors such as age, sex, medication, diet, and gut microbiome can affect the bioavailability and bioactivity of flavonoids [29]. Most flavonoids are harmless, but when taken in excess can prove to be dangerous. The side effects mentioned are gastrointestinal symptoms, hemolytic anemia, increased risk of hepatotoxicity, toxic drug interactions, contact dermatitis, and estrogen-related issues in men and breast cancer. These adverse effects are seen in patients with the long-term use of flavonoids [31].

The review by Liskova et al. [10] also touches upon another very important aspect, i.e., combinational therapy for cancer. Flavonoids, when added to other cancer therapies, have been shown to improve their efficacy. Docetaxel (DTX) is a chemotherapeutic used in metastatic cancers; however, its effect can be reduced due to phosphoinositide 3-kinase/protein kinase B (PI3K/Akt). Quercetin can inhibit the effect of PI3K/Akt, leading to the inhibition of cell migration and invasion and increasing drug buildup in the tumor, ultimately increasing the efficacy of DTX. This combination has proven to be effective in highly metastatic breast cancer cells [32]. Gemcitabine is used to treat patients with metastatic pancreatic cancer. EGCG, in its similar action to quercetin, shows an evident decrease in cancer cell growth, migration, and invasion when used in combination with gemcitabine [33]. Cyanidins showed potential to increase the efficacy of oxaliplatin, a chemotherapeutic, by reversing its resistance and increasing cell sensitivity to the drug in hepatic cellular carcinoma [34].

A large amount of clinical research have been conducted to test the efficacy of flavonoids, as discussed by Liskova et al. [10]. A phase I/II study tested the combination of genistein with FOLFOX and FOLFOX-Bevacizumav, and this was found to be safe and highly tolerable in metastatic colorectal cancer patients. Another phase II study evaluated the use of genistein in prostate cancer patients 1 month before prostatectomy and the results showed a decrease in tumor invasion [35]. EGCG had an increased bioavailability when greenselect phytosome was added to the treatment regimen in patients with early breast cancer one month before surgery [36]. Inflammation worsens metastasis and therapy resistance, and a study showed that fisetin decreased inflammation when administered 1 week before chemotherapy in colorectal carcinoma patients [37].

To conclude, flavonoids, comprising several family members with potent anticancer potential, have shown themselves to have a very promising future in cancer therapy and in the reduction in metastasis, as evidenced by research conducted in recent decades. This is the subject of discussion in the review article ‘Flavonoids in Cancer Metastasis’ by Liskova et al. [10]. As pointed out, much progress has been made, but much more research is needed to fully understand the exact and epigenetic mechanism of action of flavonoids, the tolerable doses, and whether use as monotherapy or in combination with chemo-/radiotherapies is effective. The side effects and toxicity of flavonoids still need to be studied more in depth.
